# The 5-HT_1A_ receptor biased agonists, NLX-204 and NLX-101, display ketamine-like RAAD and anti-TRD activities in rat CMS models

**DOI:** 10.1007/s00213-023-06389-5

**Published:** 2023-06-13

**Authors:** Mariusz Papp, Piotr Gruca, Magdalena Lason, Ewa Litwa, Adrian Newman-Tancredi, Ronan Depoortère

**Affiliations:** 1 Maj Institute of Pharmacology, Polish Academy of Sciences, Krakow, Poland; 2 Neurolixis SAS, 81100 Castres, France

**Keywords:** Serotonin, 5-HT_1A_ receptors, Biased agonist, Chronic mild stress, Depression, Ketamine, RAAD, TRD, Wistar-Kyoto rat, Cognition

## Abstract

**Objectives:**

NLX-101 and NLX-204 are highly selective serotonin 5-HT_1A_ ‘biased’ agonists, displaying potent and efficacious antidepressant-like activity upon acute administration in models such as the forced swim test.

**Methods:**

we compared the effects of repeated administration of NLX-101, NLX-204 and ketamine in the chronic mild stress (CMS) model of depression, considered to have high translational potential, on sucrose consumption (anhedonia measure), novel object recognition (NOR; working memory measure) and elevated plus maze (EPM; anxiety measure) in male Wistar and Wistar-Kyoto rats (the latter being resistant to classical antidepressants).

**Results:**

in Wistar rats, NLX-204 and NLX-101 (0.08–0.16 mg/kg i.p.), like ketamine (10 mg/kg i.p.) dose-dependently reversed CMS-induced sucrose intake deficit from treatment Day 1, with nearly full reversal observed at the higher dose at Days 8 and 15. These effects persisted for 3 weeks following treatment cessation. In the NOR test, both doses of NLX-101/NLX-204, and ketamine, rescued the deficit in discrimination index caused by CMS on Days 3 and 17; all three compounds increased time spent in open arms (EPM) but only NLX-204 achieved statistical significance on Days 2 and 16. In Wistar-Kyoto rats, all 3 compounds were also active in the sucrose test and, to a lesser extent, in the NOR and EPM. In non-stressed rats (both strains), the three compounds produced no significant effects in all tests.

**Conclusions:**

these observations further strengthen the hypothesis that biased agonism at 5-HT_1A_ receptors constitutes a promising strategy to achieve rapid-acting/sustained antidepressant effects combined with activity against TRD, in addition to providing beneficial effects against memory deficit and anxiety in depressed patients.

**Supplementary Information:**

The online version contains supplementary material available at 10.1007/s00213-023-06389-5.

## Introduction

Around 280 million people affected worldwide according to the World Health Organization (WHO 2011), depression remains a major health challenge in developed countries. The majority of commercially available antidepressant drugs target inhibition of monoamine neurotransmitter (mostly serotonin and noradrenaline) reuptake into brain neurons (Stahl et al. 2005). However, this approach is sub-optimal, since a substantial proportion of patients (about a third) fail to respond adequately to treatment (a phenomenon known as ‘treatment resistant depression’: TRD), or show only partial responses. Additionally, current antidepressants usually require several weeks of administration before being efficacious, and this protracted delay in therapeutic onset constitutes a source of distress to families, increased medical burden and a potential suicide risk for patients (Witkin et al. 2018).

Consequently, there has been a major effort by the drug discovery community to seek new classes of antidepressants that would overcome these drawbacks, in particular the delayed onset of activity and the treatment resistance. Thus, the glutamatergic N-methyl-D-aspartic acid (NMDA) receptor antagonist ketamine, originally developed as a general anaesthetic, is active in acute and chronic pre-clinical models of depression such as the learned helplessness, forced swim test (FST) and chronic mild stress (CMS) (Koike et al. 2011; Maeng et al. 2008). Ketamine has also demonstrated antidepressant activity in clinical trials, where it displayed rapid-acting antidepressant (RAAD) activity (within 40 min) that lasted several days following a single intravenous administration. Further, ketamine can bring relief in profoundly-depressed and in TRD patients, resulting in a reduction of suicide risk (Gould et al. 2019).

Unfortunately, ketamine is far from being an ideal antidepressant and can elicit a variety of side-effects ranging from mild to possibly life-threatening, including abuse potential, severe psychotomimetic effects, lower tract urinary problems, increased heart rate and blood pressure. These side-effects seriously limit its use (Mason et al. 2010; Niciu et al. 2018; Riva-Posse et al. 2018), and it is restricted to hospital-supervised patients in conjunction with an oral antidepressant. Secondly, ketamine is also extensively metabolized, being a substrate for CYP3A4, CYP2B6, and CYP2C9 enzymes, raising concerns of potential pharmacokinetic drug interactions (Hijazi and Boulieu 2002). Nonetheless, the enantiomer s-ketamine (Esketamine, Spravato®), under the form of a nasal spray, has been developed for treatment-resistant depression (Lener et al. 2017; Rakesh et al. 2017; Serafini et al. 2014), and was approved by the US Food and Drugs Administration (FDA) in March 2019.

Nevertheless, in view of the RAAD and TRD activities of ketamine, there is considerable interest in elucidating its underlying neurochemical mechanisms of action; these have notably focused on glutamate/AMPA neurotransmission enhancement, activation of the mTOR pathway, synaptogenic effects and pro-BDNF activity (Duman et al. 2016). Interestingly, recent pre-clinical data have pointed towards an important role of the 5-HT_1A_ receptor in ketamine’s pharmacological effects. Hence, stimulation of 5-HT_1A_ receptors in the medial prefrontal cortex (mPFC) has been proposed to mediate the antidepressant effects of ketamine in the FST in mice (Ago et al. 2022; Fukumoto et al. 2018). More precisely, ketamine exerts antidepressant-like effects that last for 24 h, and that are blocked by local micro-injection into the mPFC of the 5-HT_1A_ receptor antagonist WAY-100,635. Furthermore, these sustained antidepressant effects are mimicked by local microinjection into the mPFC, but not by systemic administration, of the 5-HT_1A_ receptor agonist, 8-OH-DPAT (Fukumoto et al. 2018). In addition to these observations, ketamine is found to elicit phosphorylation of ERK in rat cortical areas or in cultured cortical neurons (Lepack et al. 2016; Pochwat et al. 2017), suggesting that this signaling pathway may mediate antidepressant activity. This is supported by the observation that reduction of ERK phosphorylation using inhibitors of mitogen-activated protein kinase (MEK) prevents the antidepressant-like effects of ketamine in rat models (Reus et al. 2014). Taken together, these observations suggest that pharmacological interventions that elicit robust activation of cortical 5-HT_1A_ receptors and ERK phosphorylation could constitute an effective and novel strategy for RAADs which, like ketamine, could show efficacy in profoundly depressed and TRD patients.

We have previously demonstrated that NLX-101, a.k.a. F15599 (Maurel et al. 2007), is an exceptionally-selective agonist that preferentially activates 5-HT_1A_ heteroreceptors in cortical regions versus 5-HT_1A_ autoreceptors in Raphe nuclei, as observed in electrophysiology, neurochemistry and brain imaging (fMRI and PET) studies (Becker et al. 2016; Llado-Pelfort et al. 2010). This first-in-class profile appears to arise from NLX-101’s marked in vitro biased agonism for ERK phosphorylation versus other signaling pathways, including cyclic AMP inhibition, receptor internalization, beta-arrestin activation or Ca^2+^ release (Newman-Tancredi 2011; Newman-Tancredi et al. 2009; Sniecikowska et al. 2019), as well as preferential activation of ERK phosphorylation ex vivo in frontal cortex versus Raphe nuclei (Haiying et al. 2017; Newman-Tancredi et al. 2009). Most notably, NLX-101 elicits highly efficacious antidepressant-like activity in the rat FST, completely abolishing immobility upon acute and repeated administration for at least 8 h (acutely) (Assie et al. 2010). This suggests that preferential activation of ERK phosphorylation via cortical 5-HT_1A_ receptors translates to marked and lasting antidepressant-like activity (Newman-Tancredi 2011; Newman-Tancredi et al. 2009). Additionally, NLX-101 exhibits robust pro-cognitive activity in a range of rodent behavioral models, suggesting that, unlike ketamine, it is free from neurotoxic and/or cognition-disrupting effects (Depoortere et al. 2010; Horiguchi and Meltzer 2012; van Goethem et al. 2015). Finally, NLX-101 has little effect on pre-synaptic 5-HT_1A_ autoreceptors in Raphe nuclei (Llado-Pelfort et al. 2010), the activation of which is associated with delayed onset of antidepressant response (Celada et al. 2013).

More recently, another high efficacy, highly selective and high potency biased agonist at 5-HT_1A_ receptors, NLX-204 (a chemical analog of NLX-101) has been developed. This provides a means to test whether RAAD activity is an inherent characteristic of 5-HT_1A_ biased agonism for ERK, and not unique to NLX-101. In vitro, NLX-204 has high affinity (pKi = 10) at 5-HT_1A_ receptors and potently activates ERK phosphorylation more than other signaling pathways (Sniecikowska et al. 2019). In vivo, it is efficaciously and potently active acutely in the rat FST model (Sniecikowska et al. 2019), and displays RAAD-like activity in two models of depression (CMS and chronic corticosterone) in mice (Gluch-Lutwin et al. 2023). However, it is not known whether NLX-204, like NLX-101 (Depoortere et al. 2019), also exhibits RAAD properties in the rat CMS depression model. The latter possesses all three dimensions of validity (construct, face and predictive), and is considered to have high translational potential (Antoniuk et al. 2018; Willner 2017a; b). Briefly, the model consists of applying a variety of mild stressors, for several hours at a time, over an extended period of several weeks. This results in anhedonia-like behavior in rats, evidenced by their decreased preference for drinking a weak sucrose solution. Anhedonia is a recognized symptom of depression (Cao et al. 2019), so reversal of sucrose consumption deficits in the CMS model is a strong indicator of potential antidepressant activity (Willner et al. 1992). As well as detecting RAAD activity, using Wistar rats, activity against TRD can also be modeled, using a strain of rats, the Wistar-Kyoto, known to be resistant to treatment with classical antidepressants like imipramine, citalopram or venlafaxine (Willner et al. 2019).

Here, we investigated the effects of systemic NLX-204, along with those of NLX-101 and ketamine, in the CMS model in Wistar and Wistar-Kyoto male rats. As well as using the consumption of sucrose as an indicator of anhedonia, we also measured the effects of the three compounds in the novel object recognition (NOR) and the elevated plus maze (EPM), two pre-clinical models of working memory and anxiety, respectively (Ennaceur and Delacour 1988; Pellow et al. 1985), traits that are also affected in depressed patients (Willner 2017a). We have previously shown that the CMS procedure causes clear deficits in both the NOR and EPM tests, and that these deficits are reversible by antidepressant drugs (Papp et al. 2016; 2017a). Particular care was taken to address the issue of the time of onset of activity: for this reason, sucrose consumption was tested from the first day following the start of drug treatment. In addition, to examine if, similarly to ketamine, effects outlast the period of treatment (Papp et al. 2017b), rats were tested for sucrose consumption once a week for 4 weeks following cessation of drug treatment. Some results have been previously reported in the form of a conference poster (Papp et al. 2021).

## Materials and methods

To maximize comparability of the present data with those of previous studies on other antidepressants, the procedures herein were the same as those described by (Papp et al. 2017b) and the NLX compounds were administered b.i.d. (“bis in die”), and ketamine q.d. (“quaque die”) (Depoortere et al. 2019; Papp et al. 2017b). For ethical and costs reasons, for each strain, a single vehicle-injected control group and a single vehicle-injected CMS group were used in common for NLX-204, NLX-101 and ketamine. In addition, the choice of the cohort size (n = 8) for each group was based on previous laboratory experience showing that this number is sufficient to obtain stable and reproducible performances, amenable to statistical analysis, and also reasonable in terms of ethical and cost considerations. Lastly, whilst clinical protocols with ketamine in depression studies most frequently involve a single i.v. administration, herein NLX compounds and ketamine were administered repeatedly (hence the choice of i.p. route) to also verify that there was no tachyphylaxis to their beneficial pharmacological effects on sucrose intake, NOR and EPM performance.

### Animals

Male Wistar rats (weighing approximately 100 g at the start of the experiment and 330 g before starting the CMS) or Wistar-Kyoto rats (approximate weights: 120 g and 315 g, respectively), were obtained from a commercial source (Charles River, Germany) and were brought into the laboratory one month before the start of the experiment. Except for the first 10 days after arrival when the animals were housed in groups of 10, they were housed singly with free access to food and water, and maintained on a 12-h light/dark cycle (lights on at 08.00) in conditions of constant temperature (22 ± 2 °C) and humidity (45 ± 5%). All procedures used conformed to the rules and principles of EEC Directive 86/609 and were approved by the Bioethical Committee at the Institute of Pharmacology, Polish Academy of Sciences, Krakow, Poland (consent N^o^ 209/2020).

### Chronic mild Stress (CMS) Procedure: sucrose intake test

After a 3-week period of adaptation to laboratory and housing conditions, the animals were trained to consume a 1% sucrose solution; training consisted of eight weekly 1 h baseline tests, in which sucrose solution was presented, in the home cage, following 14 h of food and water deprivation. Subsequently, sucrose consumption was monitored once weekly, under similar conditions, throughout the duration of the study.

On the basis of their sucrose intakes in the 8^th^, i.e., final baseline test, the animals were divided into two groups (CMS and no CMS), matched for their sucrose consumption, such that each group had similar number of high (> 15 g), medium (11–15 g) and low (< 11 g) sucrose solution drinkers, so that the average sucrose intakes for the two groups were roughly equal. Each group was subjected to the CMS procedure for a period of 9 consecutive weeks. Each week of the stress regime consisted of two periods of food or water deprivation, two periods of 45-degree cage tilt, two periods of intermittent illumination (light on and off every 2 h), two periods of soiled cage (250 ml water in sawdust bedding), one period of paired housing, two periods of low intensity stroboscopic illumination (150 flashes/min), and three periods of no stress. All the stressors were of 10–14 h duration and were applied continuously, day and night. Control non-stressed animals were housed in separate rooms and had no contact with the stressed animals. They were deprived of food and water for 14 h before each sucrose test, but otherwise food and water were available at libitum.

Again, on the basis of their sucrose intake scores following the initial two weeks of stress, animals from both the stressed and control groups were further sub-divided (see above) into matched subgroups (n = 8 rats per subgroup). For the subsequent 17 days they received twice-daily i.p. administration of either vehicle (distilled water, 1 ml/kg), or NLX-101 or NLX-204 (0.04 or 0.16 mg/kg) at approximately 10.00 and 17.00 h each day, starting at Day 0. Ketamine (10 mg/kg i.p.) was administered once a day at approximately 10 am each day. The 3 weekly sucrose consumption tests (Days 1, 8 and 15), the EPM (Days 2 and 16) and the NOR (Days 3 and 17) tests were carried out in the mornings, 17 h following the last injection. Morning doses were administered right after completion of the various behavioral tests (see schematic in supplementary Fig. 1). After 17 days, drug administration was terminated but sucrose intake tests were conducted once a week, for another four weeks of withdrawal. CMS conditions were continued throughout the entire period of treatment and withdrawal. On the night before behavioral tests, animals received tilting or no stress; after the tests, scheduled stressors were applied (see Supplementary Fig. 1.).

### Elevated Plus Maze (EPM)

The animals were tested in two non-transparent boxes, which consisted of two open (50 × 11, L x W cm) and two closed (50 × 11 × 40, L x W x H cm) arms. The apparatus was elevated 50 cm above the floor and was illuminated by two 25 W bulbs located beneath each of the open arms. The animals were individually placed in the center of the apparatus and the number of entries into, and the time spent in the open and closed arms was manually recorded (precision ± 0.1 s) during a 5-min test, through a mirror located 1.5 m above the apparatus. An arm entry was defined as a rat having all four paws in the arm.

### Novel Object Recognition (NOR)

The animals were tested in two non-transparent open fields (100 cm in diameter, 35 cm high, with the floor divided into painted 16-cm squares). Following two 10-min adaptation sessions each during two successive days, the animals were allowed on the 3^rd^ day to explore two identical objects (white wooden cylinders, 7 cm in diameter, 11 cm high) for the time required to complete 15 s of exploration of both objects (i.e., sitting in close proximity to the objects, sniffing or touching them) (T1 session). In a retention trial conducted one hour later (T2 session), one of the objects presented previously was replaced by a novel object (black wooden prism, 5 cm wide, 14 cm high), and the duration of exploration of each object was measured during a 5 min test. A NOR index was calculated according to the following formula:

$$\frac{\left(\mathrm{time\;of\;novel\;object\;exploration}\right)-\left(\mathrm{time\;of\;familair\;object\;exploration}\right)}{\left(\mathrm{time\;of\;novel\;objects\;exploration}\right)+\left(\mathrm{time\;of\;familair\;objects\;exploration}\right)}$$

During the T2 NOR sessions, the number of line crossings was recorded as a measure of locomotor activity. All NOR parameters were recorded manually by use of a mirror located 1.5 m above the apparatus.

### Drugs

NLX-101 (also known as F15599; 3-chloro-4-fluorophenyl)-[4-fluoro-4-[[(5-methylpyrimidin-2-yl)methylamino]methyl]piperidin-1-yl]methanone, fumarate salt) and NLX-204 (3-Chloro-4-fluorophenyl)(4-fluoro-4-(((2-(pyridin-2-yloxy)ethyl)-amino)methyl)piperidin-1-yl)methanone, fumarate salt, were synthesized by Neurolixis. Racemic ketamine HCl was obtained commercially from Biowet Pulawy (Poland). Doses are expressed as the weight of the free base. The compounds were dissolved in their vehicle (distilled water), and administered i.p. in a volume of 1 ml/kg.

### Data analysis

Details of statistical analysis are available in the “Supplementary statistics” file. Two-way ANOVAs were followed by Holm-Sidak’s post-hoc tests wherever appropriate and reported by statistical symbols in figures. Statistical analyses were implemented with the Sigmastat® V4.0 or the Prism® V9.4.1 softwares.

## Results

### NLX-204 and NLX-101, like ketamine, rapidly reverse the decrease of sucrose intake induced by CMS in Wistar rats

Out of 120 Wistar and 110 Wistar-Kyoto rats originally included in the study, 24 (20%) and 14 (13%), respectively, were excluded because of either unstable drinking during the baseline phase and/or unstable and/or insufficient sucrose intake reduction during the initial two weeks of stress (i.e., before first drug dosing).

At the end of the sucrose consumption adaptation/training period, i.e. before starting the CMS procedure, baseline intake values for the 12 groups ranged from 12.4 ± 0.7 to 14.7 ± 1.6 g (mean ± SEM) and were not significantly different from one another (one-way ANOVA: F(11,84) = 0.62, p = 0.81).

**Fig. 1:**
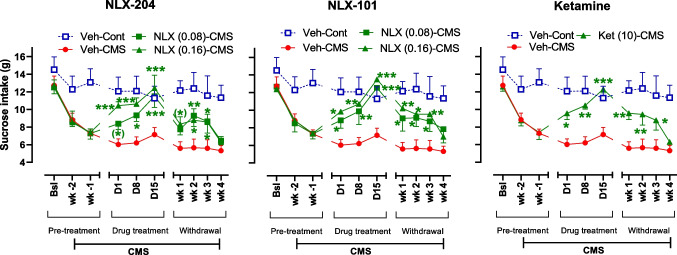
Effects of NLX 204, NLX-101 and ketamine on sucrose intake in Wistar rats subjected to CMS Sucrose intake (g) in control (Cont) and stressed (CMS) animals is shown at baseline (Bsl), during the initial two weeks of stress, i.e., before the treatments were commenced (Week-1, Week-2), during the treatment period (Days 1 to 15) and during the withdrawal period (Weeks 1 to 4). (*)*P* = 0.05, *P < 0.05, **P < 0.01, ***P < 0.001 versus CMS-Vehicle at the considered epoch, Holm-Sidak’s post-hoc test following significant two-way ANOVAs. Note that the Vehicle-Control group data are not included in the statistical analysis. For all panels, symbols/bars are means with s.e.m. Note that for the sake of clarity, only pertinent and significant post-hoc paired comparisons are shown. For all panels: see “Supplementary statistical data” file for details; *N* = 8 per group

For each of the 3 compounds, three-way ANOVAs revealed significant stress, treatment and time effects, and various interactions, depending on the compound (see “Supplementary statistic” file for details). The control, non-stressed animals administered vehicle (blue squares, Fig. 1) consumed around 12–14 g of sucrose during the 10 test sessions (baseline, pre-treatment Weeks 1 and 2, treatment Days 1, 8 and 15, and withdrawal Weeks 1–4). The stressed rats injected with vehicle (red circles) displayed a time-dependent decrease in sucrose consumption, with values ranging from about 6 to 7 g from treatment (Days 1, 8 and 15) to withdrawal (Weeks 1–4, during which the CMS was maintained) periods.

NLX-204, NLX-101 and ketamine produced similar and very rapid effects on sucrose consumption in stressed rats, with time-dependent increases in sucrose intake that were significant right from Day 1 (green symbols). For all 3 compounds, sucrose intake was nearly indistinguishable from that of the vehicle/control group on Day 15. Values for the 0.08 mg/kg dose of NLX-101 and NLX-204 were somewhat lower than those for the higher dose (0.16 mg/kg).

Upon cessation of drug treatment (withdrawal period), sucrose intake of stressed rats remained significantly higher than those of vehicle-treated stressed rats up to week 3 of withdrawal.

In non-stressed animals, for all three compounds the sucrose intake values were not significantly different from those of the vehicle-injected group (Supplementary Fig. 2).

We also verified that 0.16 mg/kg of NLX-101 and NLX-204, administered just once a day (q.d.) were also active (supplementary Fig. 4), thus showing that the effects of the NLX compounds do not require twice-daily administration.

### Differential effects of NLX-204, NLX-101 and ketamine against the increased anxiety produced by CMS in the elevated plus maze model in Wistar rats

**Fig. 2:**
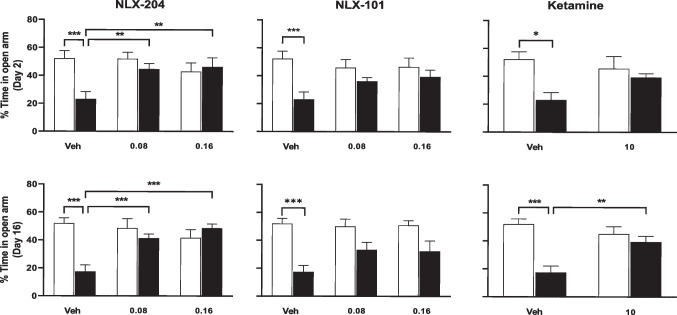
Effects of NLX 204, NLX-101 and ketamine on anxiety (EPM test) in Wistar rats subjected to CMS Percentage of time spent in the open arm at Day 2 (upper row) or at Day 16 (lower row) of drug treatment. *P < 0.05, **P < 0.01, ***P < 0.001, Holm-Sidak’s post-hoc test following significant two-way ANOVA. For all panels, symbols/bars are means with s.e.m.. Note that for the sake of clarity, only relevant and significant post-hoc paired comparisons are shown. For all panels: see “Supplementary statistical data” file for details; *N* = 8 per group

In control rats treated with vehicle, the % of time spent in the open arms on Day 2 of treatment was 52.2 ± 5.4%; rats subjected to CMS spent less than half as much time (23.1 ± 5.2%: compare, foremost left pairs of bars, upper row of Fig. 2). NLX-204 significantly modified this parameter, with the % of time in open arms of CMS rats remaining significantly higher than that of control ones at both doses. Although NLX-101 and ketamine elicited a numerical increase in the % time spent in the open arms, this did not achieve statistical significance, except for Day 16 with ketamine. None of the three compounds had significant effects in control rats (see “Supplementary statistics” file).

### NLX-204, NLX-101 and ketamine reverse the deficit in working memory produced by CMS in the novel object recognition (NOR) model in Wistar rats

**Fig. 3:**
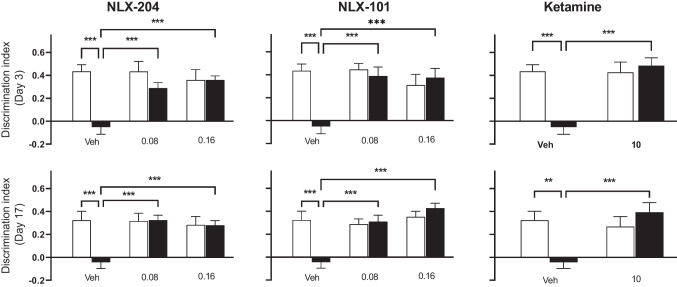
Effects of NLX 204, NLX-101 and ketamine on the discrimination index (NOR test) in Wistar rats subjected to CMS Novel versus familiar object discrimination index measured at Day 3 (upper row) or at Day 17 (lower row) of drug treatment; **P < 0.01, ***P < 0.001, Holm-Sidak’s post-hoc test following significant two-way ANOVA. For all panels, symbols/bars are means with s.e.m.. Note that for the sake of clarity, only relevant and significant post-hoc paired comparisons are shown. For all panels: see “Supplementary statistical data” file for details; *N* = 8 per group

Animals not subjected to CMS and treated with vehicle presented a NOR index around 0.4 on Day 3 of the treatment period, meaning that they explored the novel object longer than the familiar one during the second presentation trial (T2, foremost left open bars, top row panels of Fig. 3). On the other hand, the NOR index in the stressed animals receiving vehicle was close to 0, indicating that they spent a similar amount of time exploring the familiar and the novel object (compare first and second bars, Fig. 3, top panels) and reflecting a loss of NOR following exposure to chronic stress. All 3 compounds significantly reversed the CMS-induced NOR deficit, with significant effects from 0.08 mg/kg for NLX-204 and NLX-101 (compare 1^st^ with 2^nd^ and 3^rd^ solid bars). In control animals, none of the 3 compounds significantly affected the NOR index (compare foremost left open bar with subsequent open bars).

When tested on Day 17 of treatment, the NOR index of control/vehicle animals was slightly lower than that of Day 3 (around 0.3 versus 0.4: foremost left open bars, bottom row panels of Fig. 3), but the index also dropped below 0 in stressed animals receiving vehicle. Similarly to Day 3, all three compounds significantly reversed this drop of NOR index in stressed rats, whilst having no significant effect in control rats.

Locomotor activity recorded during the second session (T2) was not significantly affected by stress or drug treatment, with values ranging from 80.4 ± 7.1 to 101.1 ± 10.4 for Day 3 and 80.9 ± 8.4 to 113.0 ± 11.2 for Day 17 (Table 1).

**Table 1:** neither NLX-101, nor NLX-204 nor ketamine affect locomotor activity during the T2 session in the NOR test in Wistar rats

Treatment	At Day 3 of treatment		At Day 17 of treatment	
	Control	CMS	Control	CMS
Vehicle	80.4 ± 7.1	91.4 ± 7.0	82.0 ± 7.0	80.9 ± 8.4
NLX-101 (0.08 mg/kg)	89.5 ± 11.6	94.3 ± 10.7	88.5 ± 11.9	104.1 ± 10.9
NLX-101 (0.16 mg/kg)	100.1 ± 10.4	83.8 ± 6.5	113.0 ± 11.2	91.4 ± 7.2
NLX-204 (0.08 mg/kg)	86.1 ± 4.3	89.5 ± 11.1	96.9 ± 11.6	112.8 ± 9.6
NLX-204 (0.16 mg/kg)	92.6 ± 11.7	71.9 ± 6.8	100.8 ± 13.0	85.9 ± 9.7
Ketamine (10 mg/kg)	94.1 ± 7.0	94.8 ± 18.3	90.6 ± 18.7	98.4 ± 14.9

Locomotor activity (number of crossings) is presented as mean ± s.e.m.. Drug-treated rats did not show significantly different locomotor activity when compared to respective vehicle-treated rats. See “Supplementary statistics” file for full details of two-way ANOVAs

### Neither NLX-204, nor NLX-101 nor ketamine affect body weight during the CMS in Wistar rats

At Day 15 of treatment, body weights ranged from 321.9 ± 4.4 to 360.0 ± 5.2 g (supplementary Table 1). At the end of the withdrawal period (Week 4), rats had gained weight, as expected, ranging from 356.3 ± 10.6 to 389.4 ± 6.0 g. None of the three compounds or stress significantly affected body weight (see “Supplementary statistics” file).

### NLX-204, similarly to NLX-101 and ketamine, also reverses the decrease of sucrose intake induced by CMS in Wistar-Kyoto rats

At the end of the sucrose consumption adaptation/training period, i.e. before starting the CMS procedure, baseline intake values for the 12 groups ranged from 12.7 ± 1.6 to 13.9 ± 1.0 g (mean ± SEM) and were not significantly different from one another (one-way ANOVA: F(11,84) = 0.07, p = 0.99).

**Fig. 4:**
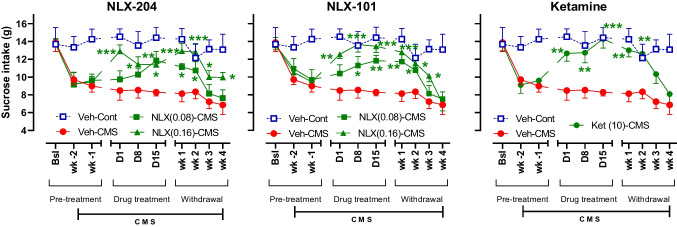
Effects of NLX 204, NLX-101 and ketamine on sucrose intake in Wistar-Kyoto rats subjected to CMS Sucrose intake (g) in control (Cont) and stressed (CMS) animals is shown at baseline (Bsl), during the initial two weeks of stress, i.e., before the treatments were commenced (Week-1, Week-2), during the treatment period (Days 1 to 15) and during the withdrawal period (Weeks 1 to 4). *P < 0.05, **P < 0.01, ***P < 0.001 versus CMS-Vehicle at the considered epoch, Holm-Sidak’s post-hoc test following significant two-way ANOVAs. Note that the Vehicle-Control group data are not included in the statistical analysis. For all panels, symbols/bars are means with s.e.m. Note that for the sake of clarity, only pertinent and significant post-hoc paired comparisons are shown. For all panels: see “Supplementary statistical data” file for details; *N* = 8 per group

For each of the 3 compounds, three-way ANOVAs revealed significant stress, treatment and time effects and various interactions, depending on the compound (see “Supplementary statistic” file for details). The control, non-stressed animals administered vehicle (blue squares in Fig. 4) consumed around 13–14 g of sucrose during each of the 10 test sessions. The stressed rats injected with vehicle (red circles) displayed a marked decrease in sucrose consumption, with values fluctuating around 7–8 g. from treatment Day 1 to withdrawal Week 4.

Following i.p. administration, NLX-204, NLX-101 and ketamine produced similar and very rapid effects on sucrose consumption in stressed rats, with significant increase in sucrose intake starting from Day 1 for the higher doses of NLX-204 and NLX-101, and for ketamine (green symbols). Sucrose intake for NLX-101 and ketamine was nearly indistinguishable from that of the control/vehicle group on Day 15. Upon cessation of drug treatment (withdrawal period), sucrose intake did not immediately return to values of vehicle-treated stressed rats but remained significantly higher for 2–4 weeks.

In control animals, NLX-204, NLX-101 and ketamine produced very mild and non-statistically significant enhancement of sucrose consumption during both the treatment and withdrawal periods (supplementary Fig. 3).

### NLX-204, NLX-101 and ketamine diminish, during Day 2, the increased anxiety produced by CMS in the elevated plus maze model in Wistar-Kyoto rats

**Fig. 5:**
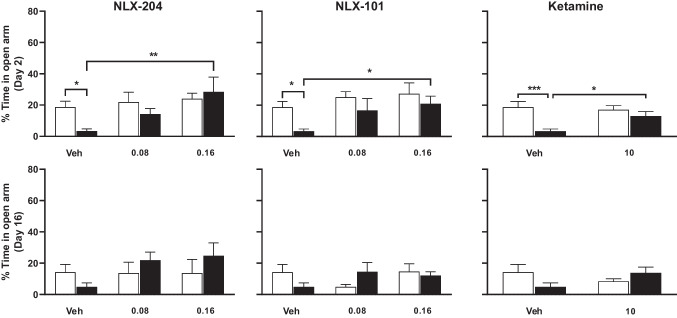
Effects of NLX 204, NLX-101 and ketamine on anxiety (EPM test) in Wistar-Kyoto rats subjected to CMS Percentage of time spent in the open arm at Day 2 (upper row) or at Day 16 (lower row) of drug treatment. *P < 0.05, **P < 0.01, ***P < 0.001, Holm-Sidak’s post-hoc test following significant two-way ANOVA. For all panels, symbols/bars are means with s.e.m.. Note that for the sake of clarity, only relevant and significant post-hoc paired comparisons are shown. For all panels: see “Supplementary statistical data” file for details; *N* = 8 per group

In control rats treated with vehicle, the % of time spent in the open arm on Day 2 of treatment was 18.7 ± 3.7%; rats subjected to CMS remained in open arms much less (3.4 ± 1.4%: compare, foremost left pair of bars, Fig. 5 upper row). All three compounds significantly reversed the CMS-induced drop in the % time; this was dose-dependent for NLX-204 and NLX-101. In contrast, on Day 16, the differential between the control/vehicle and the CMS/vehicle groups was less than that for Day 2 (14.2 5.1 ± versus 4.8 ± 2.6%) and was non significant, and only NLX-204 showed a tendency to increase the % of time spent in the open arm.

### NLX-204, NLX-101 and ketamine reverse the deficit in working memory produced by CMS in the novel object recognition (NOR) model in Wistar-Kyoto rats

**Fig. 6:**
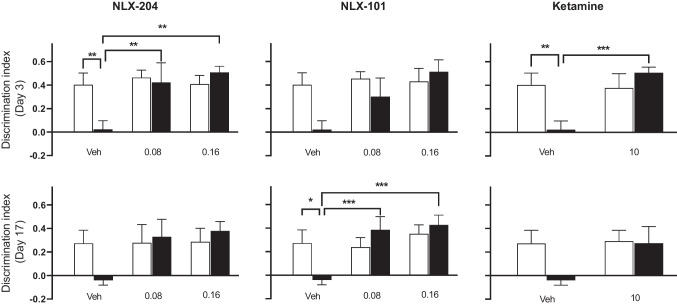
Effects of NLX 204, NLX-101 and ketamine on the discrimination index (NOR test) in Wistar-Kyoto rats subjected to CMS Novel versus familiar object discrimination index measured at Day 3 (upper row) or at Day 17 (lower row) of drug treatment; *P < 0.05, **P < 0.01, ***P < 0.001, Holm-Sidak’s post-hoc test following significant two-way ANOVA. For all panels, symbols/bars are means with s.e.m.. Note that for the sake of clarity, only relevant and significant post-hoc paired comparisons are shown. For all panels: see “Supplementary statistical data” file for details; *N* = 8 per group

Control animals receiving vehicle presented a NOR index around 0.4 on Day 3, which dropped to around 0 in CMS/vehicle rats (compare first and second bars, Fig. 6, top row). All three compounds significantly reversed (or showed a strong tendency for NLX-101) the CMS-induced NOR deficit. On Day 17, the index for the Cont/vehicle animals was lower at around 0.25, and dropped close to 0 for stressed rats. Only NLX-101 significantly reversed the CMS-induced index reduction, with the other two compounds only showing a tendency on Day 17. For both Days, none of the three compounds affected the index in control animals.

Locomotor activity recorded during the second session (T2) during treatment Day 3 ranged from 44.4 ± 5.9 to 66.8 ± 7.5, but was not significantly affected by stress or drug treatment. For Day 17, the range was from 43.3 ± 3.1 to 80.4 ± 6.3, with significant treatment effects for NLX-204 at 0.16 mg/kg under stress (Table 2).

**Table 2:** neither NLX-101, nor NLX-204 nor ketamine affect locomotor activity during the T2 session in the NOR test in Wistar-Kyoto rats

Treatment	At Day 3 of treatment		At Day 17 of treatment	
	Control	CMS	Control	CMS
Vehicle	66.8 ± 7.5	54.1 ± 7.8	64.4 ± 5.9	80.4 ± 6.3
NLX-101 (0.08 mg/kg)	63.3 ± 6.1	60.1 ± 6.6	61.9 ± 3.7	67.1 ± 12.6
NLX-101 (0.16 mg/kg)	64.9 ± 3.8	58.6 ± 6.4	60.4 ± 6.2	58.4 ± 6.4
NLX-204 (0.08 mg/kg)	60.6 ± 7.9	54.0 ± 7.2	43.3 ± 3.1	65.6 ± 4.5
NLX-204 (0.16 mg/kg)	51.0 ± 4.3	44.4 ± 5.9	46.4 ± 3.7	57.4 ± 6.6*
Ketamine (10 mg/kg)	64.6 ± 6.2	54.5 ± 4.7	50.0 ± 6.9	68.3 ± 4.9

Locomotor activity (number of crossings) is presented as mean ± s.e.m.. Drug-treated rats did not show significantly different locomotor activity when compared to respective vehicle-treated rats. See “Supplementary statistics” file for full details of two-way ANOVAs. *:* P < 0.05*, versus corresponding vehicle

### Neither NLX-204, nor NLX-101 nor ketamine affect body weight during the CMS in Wistar-Kyoto rats

At Day 15 of treatment, body weights ranged from 291.9 ± 4.5 to 323.1 ± 7.8 g, and only ketamine significantly affected weight (but no significant difference between groups in post-hoc tests: supplementary Table 2). At the end of the withdrawal period (Week 4), rats had gained some weight, as expected, ranging from 316.3 ± 5.4 to 351.3 ± 6.7 g. None of the three compounds significantly affected body weight (see “Supplementary statistics” file). For both Days, stress significantly affected body weight for all three compounds.

## Discussion

We describe, for the first time, a comparison of the effects of the 5-HT_1A_ receptor biased agonists, NLX-101 and NLX-204, with ketamine on anhedonia and other behavioral deficits in the CMS model. There were several notable findings: (i) In Wistar rats, NLX-101 and NLX-204, like ketamine, exhibited potent, complete and rapid anti-anhedonic effects, attenuating the CMS-induced deficit in sucrose consumption from Day 1 of treatment and fully reversing it at Day 15. (ii) The attenuation of sucrose consumption deficit by all three compounds persisted for 3 weeks after cessation of treatment, suggesting lasting remodeling of circuitry controlling anhedonia / depression. (iii) All 3 compounds also fully reversed CMS-induced deficit in working memory, as assessed in the NOR model, but only NLX-204 was significantly efficacious against CMS-induced anxiety behavior, as assessed in the EPM model. (iv) In Wistar-Kyoto rats, a strain resistant to classical antidepressant treatment, all three compounds were active in the sucrose consumption test, similarly to observations in Wistar rats, and were also protective against working memory deficits, but showed less anxiolytic efficacy.

### Rapid and complete anti-anhedonic activity of NLX-204, NLX-101 and ketamine in Wistar rats

At the dose of 0.16 mg/kg i.p., b.i.d., both NLX-204 and NLX-101 fully rescued the decrease of sucrose intake induced by CMS in Wistar rats: a robust attenuation of the deficit was observed right from Day 1 (i.e., the day after starting treatment, about 17 h following the second i.p. administration), with almost complete reversal observed on the subsequent test days (i.e., Days 8 and Day 15). A lower dose of both NLX compounds (0.08 mg/kg) also elicited a partial rescue of sucrose consumption on Day 1, with a larger effect on Days 8 and 15. Strikingly, the rapid and efficacious effects of NLX-101 and NLX-204 were very similar to those obtained with ketamine (10 mg/kg, i.p., q.d.) and suggests that the three compounds may display similar RAAD activity.

The present data on NLX-101 (0.16 mg/kg i.p., b.i.d.) are generally consistent with those in our previous publication on this compound (Depoortere et al. 2019), with NLX-101 eliciting a rapid (from Day 1) and full (on Days 1–15) rescue of anhedonia in the sucrose intake test, and a clear maintenance of anti-anhedonic activity beyond treatment withdrawal.

More generally, the present data for NLX-204 extend its profile of activity in preclinical models of depression. The compound is potently and efficaciously active upon acute administration in the FST in naïve rats (Sniecikowska et al. 2019), and displays RAAD-like activity in the CMS and chronic corticosterone assays in mice (Głuch-Lutwin et al. 2021).

Of note, as well as increasing pERK1/2 levels in PFC of CMS mice, NLX-204 also increased pCREB levels in the PFC of corticosterone-treated mice, suggesting that this may constitute an additional pathway involved in RAAD effects of 5-HT_1A_ receptor biased agonists.

In the present study, ketamine exhibited a RAAD-like profile similar to that observed for NLX-204 and NLX-101, with partial but significant reversal of CMS-induced sucrose intake decrease right from Day 1 and full reversal at Day 15, followed by sustained activity upon drug withdrawal. In contrast, in a previous study using a similar procedure in Wistar rats (Papp et al. 2017b), ketamine (10 mg/kg i.p.), only elicited significant (and partial) anti-anhedonic activity from Day 8, and a treatment duration of two weeks was necessary for ketamine to achieve near-complete anti-anhedonic activity. It is unclear why ketamine was found to be more rapidly active on sucrose consumption in the present study, but the effects of ketamine on CMS-induced NOR index reduction, as well as attenuated anxiety (EPM model) in CMS rats were similar to those reported previously (Papp et al. 2017b).

### NLX-204, NLX-101 and ketamine display rapid and complete anti-anhedonic activity in Wistar-Kyoto rats

Wistar-Kyoto rats are resistant to classical antidepressants and are considered to be useful to model TRD (Aleksandrova et al. 2019; Willner et al. 2019). Both NLX compounds, in this strain of rats, displayed an anti-anhedonic profile of activity reminiscent of that in the Wistar strain, i.e., it was rapid, complete and persisted beyond termination of administration. To the best of our knowledge, this is the first demonstration that activation of 5-HT_1A_ receptors provides relief in the Wistar-Kyoto rats model of TRD. Such an activity adds an extra dimension to the pharmacological impact of NLX-204 and NLX-101, in the sense that they could represent not only a new class of RAAD, but also be active against TRD, similarly to ketamine. The present results also buttress those of a previous study in Wistar-Kyoto rats where ketamine (10 mg/kg i.p.) partly reversed CMS-induced sucrose intake deficit within 1 day of treatment (around 50% effect) (Willner et al. 2019). Ketamine has also shown RAAD-like activity in various mouse protocols of the CMS paradigm (Ramaker and Dulawa 2017).

### Sustained anti-anhedonic activity of NLX-204, NLX-101 and ketamine after cessation of treatment in both strains of rats

The anti-anhedonic effect of the three compounds was not only maintained throughout the period of treatment (as seen in the last sucrose consumption test on Day 15) but, remarkably, outlasted it, i.e., it was also observed, albeit with a more modest magnitude, during the withdrawal period, when CMS continued but the compounds were no longer administered. Indeed, sucrose intake for compound-treated rats remained significantly above that of vehicle-treated rats for 2 to 4 weeks, depending on the strain/drug combination. Importantly, NLX-101 and NLX-204 have a rat plasma half-life in rat of about 2 h, and the former has no known active metabolites and is rapidly washed out (unpublished results). Ketamine has even a shorter rat plasmatic half-life, around 1.3 h (Veilleux-Lemieux et al. 2013), although it is possible that active metabolite(s) (i.e., norketamine) might slightly extend the anti-anhedonic effects. In any case, the protracted post-withdrawal anti-anhedonic activities of NLX-101, NLX-204 and ketamine cannot be readily attributed to pharmacokinetic properties, but likely stem from downstream effects on biochemical processes such as enhanced BDNF production and/or neuronal plasticity (discussed below).

In their previous study, Papp and co-workers observed that both ketamine and imipramine (10 mg/kg i.p. each) maintained their anti-anhedonic activity for one week following withdrawal of drug administration (Papp et al. 2017b), an effect that may involve cortical–ventral hippocampal interactions (Carreno et al. 2016). Although testing was not continued beyond a single week, this suggests that antidepressants of different pharmacological classes, including 5-HT_1A_ agonists, may elicit lasting changes in sucrose consumption responses and, correspondingly, in their anti-anhedonic effects.

### Beneficial activity of NLX-204, NLX-101 and ketamine against deficit of working memory in the NOR test in both strains

In Wistar rats, all three compounds ameliorated the CMS-induced impairment of discrimination index in the NOR model, at Day 3 and Day 17, suggesting that they reversed the cognitive deficit produced by CMS. This model is thought to provide a measure of working memory, which can be seriously impaired in depressed patients (Chai et al. 2018). Of note, none of the compounds were significantly active when tested in non-stressed rats, and locomotor scores recorded during T2 were not affected by stress or treatment. Hence, the increase in the time exploring the new object in stressed rats during T2 is unlikely to result from a non-specific enhancement of locomotor activity or memory capacity per se. The previous publication on NLX-101 reported NOR results similar to the present ones and the compound has been shown to have beneficial effects in a variety of rodent cognition/memory models (see (Newman-Tancredi et al. 2022a) for extensive review). In summary, the effects of NLX-101, NLX-204 and ketamine in these various models suggests that they have a similar capacity to improve cognition/memory dysfunction associated with depressive states. Another information from these NOR data is that the efficacy of all three compounds in restoring a normal index in CMS rats is preserved between Day 3 and Day 17, suggesting a lack of tachyphylaxis.

### Variable effects of NLX-204, NLX-101 and ketamine against anxiety-like state in the EPM model in both strains

NLX-204 displayed a robust anxiolytic-like effect, both at Day 2 and Day 16 of treatment in Wistar rats subjected to CMS, and at Day 2 in Wistar-Kyoto. NLX-101, on the other hand, was not significantly active in the Wistar strain, but displayed anxiolytic-like profile in the Wistar-Kyoto strain on Day 2. The reasons for this differential anxiolytic-like activity between the two NLX compounds in both strains are not clear at present, but it might be that they differ subtly in their biased agonism profile. Results for NLX-101 in the Wistar strain are, on the whole, congruent with those of a previous study (Depoortere et al. 2019). The differential effects of NLX-101 and NLX-204 as a function of the rat strain is more puzzling, and would deserve further investigation. Regarding ketamine, the present results are on the whole in agreement with those of previous studies in Wistar (Papp et al. 2017b) or Wistar-Kyoto rats, even though the effects in the former strain on Day 2 missed significance.

### Potential mechanisms underlying NLX-101 and NLX-204 activities

Firstly, the effects of NLX-101 and NLX-204 can be confidently attributed to specific activation of 5-HT_1A_ receptors, in view of their exceptional selectivity for 5-HT_1A_ receptors in vitro (over 1000-fold selectivity against 50 targets, including most common monoaminergic receptors: data on file and (Newman-Tancredi et al. 2022a). Furthermore, the in vivo effects of NLX-101 in other models, at the doses used herein, are abolished or greatly attenuated by co-administration of the selective 5-HT_1A_ receptor antagonist WAY-100,635 (Assie et al. 2010; Jastrzebska-Wiesek et al. 2018; Llado-Pelfort et al. 2010; van Goethem et al. 2015; van Hagen et al. 2022). Lastly, microinjection of WAY-100,635 in the frontal cortex abolishes systemic effects of NLX-101 and NLX-204 in the sucrose intake, NOR and EPM models in CMS rats (Newman-Tancredi et al. 2022b). It can therefore be concluded that the pharmacological effects of NLX-101 and NLX-204 observed in the present CMS model are solely consecutive to activation of 5-HT_1A_ receptors.

Secondly, it is known that systemically administered NLX-101 increases the electrical activity of pyramidal neurons in the frontal cortex. This is likely to be consequent to preferential activation by NLX-101 of 5-HT_1A_ heteroreceptors expressed on cortical GABAergic inhibitory interneurons, thus reducing release of GABA and disinhibiting pyramidal cortical glutamatergic neurons (Llado-Pelfort et al. 2010, 2012). Such a mechanism of action is analogous to that of ketamine which, by blockade of NMDA receptors on GABAergic interneurons, also disinhibits the activity of pyramidal neurons in the cortex. It is therefore possible that NLX-101 and ketamine possess converging signaling mechanisms, a conclusion which is reinforced by the observation that, as mentioned in the Introduction, both compounds elicit phosphorylation of ERK in cortical regions (Li et al. 2010; Newman-Tancredi et al. 2009; Reus et al. 2014). This may be important for their antidepressant-like activity because ERK phosphorylation deficits are associated with depressed mood (see (Depoortere et al. 2019) for more in depth discussion). Taken together, these observations suggest that the RAAD profile of NLX-101 and NLX-204 are mediated by efficacious inhibition of cortical GABAergic interneurons, disinhibition of glutamatergic pyramidal neurons and consequent activation of AMPA receptors and ERK phosphorylation in post-synaptic cortical neurons (see Fig. 6 in (Depoortere et al. 2019)). Indeed, microinjection of the GABA_A_ receptor agonist muscimol in the frontal cortex abolishes systemic effects of NLX-101 and NLX-204 in the sucrose intake, NOR and EPM models in CMS rats (Newman-Tancredi et al. 2022b).

Thirdly, the protracted anti-anhedonic effects of the three compounds, between 2 to 4 weeks following cessation of treatment, suggest that they induce long-lasting remodeling of neuronal architecture, as has already been suggested for ketamine (Phoumthipphavong et al. 2016). When NLX-101 was administered to rats for 14 days at the same dose as that used herein (0.32 mg/kg/day i.p.), it was found to increase BDNF levels and the density of doublecortin-positive neurons in hippocampi, an indication of enhanced neurogenesis (van Hagen et al. 2022). Interestingly, the density of 5-HT_1A_ receptors was unchanged in hippocampi but downregulated in the dorsal Raphe nucleus, suggesting that there may be an increase in serotonergic tone in projection regions. Such a regulatory effect may underlie the sustained anti-anhedonic effects of NLX-101 and NLX-204.

The above considerations suggest that on one hand, the acute effects of these compounds in models of depression such as the FST, and the very rapid anti-anhedonic response to the compounds (i.e., Day 1) seen in the present CMS model are mediated indirectly by increased cortical glutamatergic tone, resulting from a lowered inhibitory action of GABAegic interneurons (see above). Interestingly, a microinjection into the frontal cortex of the GABA_A_ receptor agonist muscimol (triggering a local increased GABAergic tone) opposed the beneficial effects of i.p. administration of NLX-101 and NLX-204 in the sucrose intake and NOR models of CMS rats (Newman-Tancredi et al. 2022b). This further reinforces the hypothesis that reduction of inhibitory GABAergic tone, resulting in augmented cortical glutamatergic tone, underlies the pharmacological effects of the NLX compounds. On the other hand, medium to longer term neurochemical/anatomical modifications (increases in BDNF release, neurogenesis and re-modeling of neuronal networks) would seem to be responsible for a sustained antidepressant activity.

### Potential mechanisms underlying NLX-101 and NLX-204 activities

Some potential confounds can be excluded: (i) the enhanced consumption of sucrose solution in CMS rats treated with NLX-204, NLX-101 or ketamine is unlikely to result from a compensatory food intake mechanism, because the weights of the rats were not significantly affected by pharmacological treatment or by CMS; (ii) locomotor activity of NLX-101-treated rats in the NOR model was not significantly different from that of vehicle-injected rats, both under control and CMS conditions (Tables 1 and 2). Also, no gross behavioral changes, such as sedation or hyperlocomotion, were observed in any of the groups of rats, so it is unlikely that the RAAD-like effects of the 3 compounds result from a non-specific behavioral activation phenomenon.

Nevertheless, the present study was conducted in male rats, and it would be informative to repeat it in female rats, given that depression affects more women than men. Also, it would be valuable to compare in a head-to-head experiment the effects of the NLX compounds of ketamine with those of a non-biased 5-HT_1A_ agonist such as 8-OH-DPAT in the CMS model in Wistar-Kyoto rats (as a reminder, 8-OH-DPAT (0.5 mg/kg, s.c., b.i.d.) was shown to be inactive in Wistar rats (Przegalinski et al. 1994)).

## Conclusions and perspectives

The search for novel RAADs and compounds active against TRD has been a major goal of neuropsychiatry drug discovery for several decades, and the CMS model is one of the most highly regarded animal models for characterizing putative new antidepressant compounds. The present data further confirm that preferential activation of cortical 5-HT_1A_ receptors, using biased agonists such as NLX-101 or NLX-204, could constitute an innovative strategy to achieve not only rapid and large-magnitude antidepressant profile (cf. RAAD activity in both Wistar and Wistar-Kyoto rats), but also efficacy against TRD (cf. activity in Wistar-Kyoto rats) analogous to that of ketamine. The present observations in the NOR and EPM models also suggest that selective targeting of 5-HT_1A_ receptors could confer supplemental benefits against memory/cognitive deficits and anxiety that can afflict depressed patients.

Indeed, there is now abundant and congruent findings pointing to the utility of activating specific 5-HT receptor subtypes (instead of non-specifically augmenting central 5-HT tone, as obtained with classical SSRIs) to combat depression. One of the major receptor subtypes that has emerged as being of most interest is the 5-HT_1A_ receptor, exemplified by NLX compounds (direct activation) and ketamine/esketamine (indirect activation). An additional advantage of selectively targeting 5-HT_1A_ receptors is an anticipated mild side-effects profile compared with that elicited by blockade of NMDA receptors (hallucinations, abuse potential, etc.…). Such considerations are pertinent in view of current debate concerning the opportunities and limitations of using ketamine/esketamine in depressed patients, notably considering potentially serious adverse events (vide supra), the necessity for strict medical supervision, and the required co-treatment with an oral classical antidepressant. Clinical studies in depressed and/or TRD patients are warranted to investigate the utility of using highly selective 5-HT_1A_ receptor biased agonists such as NLX-204 or NLX-101 in such patient populations.

### Supplementary Information

Below is the link to the electronic supplementary material.

Supplementary file1 (DOCX 346 KB)

Supplementary file2 (DOCX 75 KB)
