# Supplementary material for: The 5-HT1A receptor biased agonists, NLX-204 and NLX-101, display ketamine-like RAAD and anti-TRD activities in rat CMS models
Source: Psychopharmacology (Berl). 2023 Jun 13;240(11):2419–33. doi: 10.1007/s00213-023-06389-5 (PMC10593613; doi:10.1007/s00213-023-06389-5)
Supplement: Supplementary file 1 — Supplementary file1 (DOCX 346 KB) [file 213_2023_6389_MOESM1_ESM.docx]

Supplementary figure 1: Timeline of chronic mild stress protocol

Rats were subjected to an initial adaptation period, followed by sucrose intake training. Rats were then divided into stress or control groups for 2 weeks before start of drug administration. Sucrose intake tests were carried out on Days 1, 8 and 15 (D1, D8, D15). Drug administration was then withdrawn, and sucrose intake tests carried out weekly (W1, W2, W3, W4). The Elevated Plus Maze test (EPM) was carried out on Days 2 and 16, the Novel Object Recognition test (NOR) was carried out on Days 3 and 17.

Supplementary figure 2: NLX-204, NLX-101 and ketamine do not modify sucrose intake in control (non-stressed) Wistar rats

Sucrose intake (g) in control (Cont) animals is shown at baseline (B), before starting CMS (Week-2, Week-1), during the treatment period (Days 1 to 15) and during the withdrawal period (Weeks 1 to 4). For all panels, symbols are means with s.e.m.. See “Supplementary statistics” file for details. N = 8 per group.

Supplementary figure 3: NLX-204, NLX-101 and ketamine do not modify sucrose intake in control (non-stressed) Wistar-Kyoto rats

See legend of supplementary figure 2 for details. For all panels, symbols are means with s.e.m.. See “Supplementary statistics” file for details. N = 8 per group.

Supplementary figure 4: NLX-204 and NLX-101 administered q.d. also reverse the stress-induced decrease of sucrose intake in Wistar rats

See legend of Supplementary figure 1 for details. For all panels, symbols are means with s.e.m.. Two-way ANOVAs: all Fs >2.63, all p<0.05, except time x treatment factor for NLX-101: F(6,60) = 2.13, ns. ^(^*^)^P = 0.06, **P < 0.01, ***P < 0.001 versus Veh/CMS at the considered epoch, Holm-Sidak’s post-hoc test following significant two-way ANOVA. N = 6 per group.

### Supplementary table 1: neither NLX-101, nor NLX-204 nor ketamine affect body weight in Wistar rats

| **Treatment** | **At Day 15 of treatment** | | **After 4 weeks of withdrawal** | |
| --- | --- | --- | --- | --- |
|  | Control (g) | CMS (g) | Control (g) | CMS (g) |
| Vehicle | 336.3 ± 10.0 | 327.5 ± 8.4 | 358.1 ± 10.1 | 356.3 ±10.6 |
| NLX-101 (0.08 mg/kg) | 341.9 ± 9.8 | 332.5 ± 7.1 | 368.8 ± 10.1 | 375.6 ± 8.0 |
| NLX-101 (0.16 mg/kg) | 336.9 ± 7.4 | 340.6 ± 9.7 | 363.8 ± 7.6 | 371.3 ± 12.3 |
| NLX-204 (0.08 mg/kg) | 350.0 ± 12.5 | 321.9 ± 4.4 | 381.3 ± 14.3 | 358.1 ± 6.5 |
| NLX-204 (0.16 mg/kg) | 343.8 ± 12.5 | 335.09 ± 7.6 | 375.0 ± 8.8 | 370.0 ± 9.7 |
| Ketamine (10 mg/kg) | 360.0 ± 5.2 | 332.5 ± 11.8 | 389.4 ± 6.0 | 361.9 ± 16.1 |

Body weights (in g) are presented as mean ± s.e.m.. Drug-treated rats did not show significantly different body weights when compared to respective vehicle-treated rats. See “Supplementary statistics” file for full details of two-way ANOVAs.

### Supplementary table 2: neither NLX-101 nor NLX-204 nor ketamine affect body weight during CMS in Wistar-Kyoto rats

| **Treatment** | **At Day 15 of treatment** | | **After 4 weeks of withdrawal** | |
| --- | --- | --- | --- | --- |
|  | Control (g) | CMS (g) | Control (g) | CMS (g) |
| Vehicle | 306.9 ± 8.8 | 291.9 ± 4.5 | 333.8 ± 7.4 | 316.9 ± 5.2 |
| NLX-101 (0.08 mg/kg) | 311.3 ± 5.8 | 305.6 ± 6.5 | 338.8 ± 7.6 | 316.3 ± 5.4 |
| NLX-101 (0.16 mg/kg) | 311.9 ± 6.1 | 305.6 ± 6.1 | 341.9 ± 5.5 | 325.6 ± 5.5 |
| NLX-204 (0.08 mg/kg) | 317.5 ± 8.6 | 296.9 ± 4.9 | 343.8 ± 9.4 | 322.5 ± 4.8 |
| NLX-204 (0.16 mg/kg) | 322.5 ± 4.8 | 298.1 ± 5.4 | 351.3 ± 6.7 | 323.8 ± 7.0 |
| Ketamine (10 mg/kg) | 323.1 ± 7.8 | 305.6 ± 5.9 | 349.4 ± 9.2 | 328.1 ± 6.4 |

Body weights are presented in g as mean ± s.e.m.. Drug-treated rats did not show significantly different body weights when compared to respective vehicle-treated rats. See “Supplementary statistics” file for full details of two-way ANOVAs.
