# Supplementary material for: The 5-HT1A receptor biased agonists, NLX-204 and NLX-101, display ketamine-like RAAD and anti-TRD activities in rat CMS models
Source: Psychopharmacology (Berl). 2023 Jun 13;240(11):2419–33. doi: 10.1007/s00213-023-06389-5 (PMC10593613; doi:10.1007/s00213-023-06389-5)
Supplement: Supplementary file 2 — Supplementary file2 (DOCX 75 KB) [file 213_2023_6389_MOESM2_ESM.docx]

**Wistar rats**

**Three Way Analysis of Variance, sucrose intake**

**Data source:** Ketamine

**Source of Variation DF SS MS F P**

Stress 1 1511.669 1511.669 134.763 <0.001

Treatment 1 197.868 197.868 17.640 <0.001

Time 9 407.758 45.306 4.039 <0.001

Stress x Treatment 1 63.894 63.894 5.696 0.018

Stress x Time 9 211.640 23.516 2.096 0.030

Treatment x Time 9 104.825 11.647 1.038 0.409

Stress x Treatment x Time 9 90.761 10.085 0.899 0.527

Residual 280 3140.824 11.217

Total 319 5729.239 17.960

**Data source:** NLX-101

**Source of Variation DF SS MS F P**

Stress 1 2082.958 2082.958 159.485 <0.001

Treatment 2 362.293 181.147 13.870 <0.001

Time 9 424.112 47.124 3.608 <0.001

Stress x Treatment 2 70.172 35.086 2.686 0.069

Stress x Time 9 330.156 36.684 2.809 0.003

Treatment x Time 18 260.908 14.495 1.110 0.339

Stress x Treatment x Time 18 57.726 3.207 0.246 0.999

Residual 420 5485.411 13.061

Total 479 9073.737 18.943

**Data source:** NLX 204

**Source of Variation DF SS MS F P**

Stress 1 2414.051 2414.051 176.679 <0.001

Treatment 2 333.412 166.706 12.201 <0.001

Time 9 511.684 56.854 4.161 <0.001

Stress x Treatment 2 51.630 25.815 1.889 0.152

Stress x Time 9 299.978 33.331 2.439 0.010

Treatment x Time 18 163.708 9.095 0.666 0.845

Stress x Treatment x Time 18 65.667 3.648 0.267 0.999

Residual 420 5738.667 13.663

Total 479 9578.799 19.997

| Two-way RM ANOVA,  **sucrose intake** |  |  |  |  |  |
| --- | --- | --- | --- | --- | --- |
|  |  |  |  |  |  |
| Ketamine, CMS |  |  |  |  |  |
|  |  |  |  |  |  |
| ANOVA table | SS | DF | MS | F (DFn, DFd) | P value |
| Time x Treatment | 148.2 | 9 | 16.46 | F (9, 126) = 6.504 | P<0.0001 |
| Time | 496.4 | 9 | 55.15 | F (9, 126) = 21.79 | P<0.0001 |
| Treatment | 244.0 | 1 | 244.0 | F (1, 14) = 9.374 | P=0.0085 |
| Subject | 364.5 | 14 | 26.03 | F (14, 126) = 10.29 | P<0.0001 |
| Residual | 318.9 | 126 | 2.531 |  |  |
|  |  |  |  |  |  |

NLX-101, CMS

| ANOVA table | SS | DF | MS | F (DFn, DFd) | P value |
| --- | --- | --- | --- | --- | --- |
| Time x Treatment | 226.9 | 18 | 12.61 | F (18, 189) = 3.055 | P<0.0001 |
| Time | 668.3 | 9 | 74.26 | F (9, 189) = 17.99 | P<0.0001 |
| Treatment | 375.2 | 2 | 187.6 | F (2, 21) = 4.732 | P=0.0201 |
| Subject | 832.6 | 21 | 39.65 | F (21, 189) = 9.606 | P<0.0001 |
| Residual | 780.1 | 189 | 4.127 |  |  |

NLX-204, CMS

| ANOVA table | SS | DF | MS | F (DFn, DFd) | P value |
| --- | --- | --- | --- | --- | --- |
| Time x Treatment | 183.0 | 18 | 10.17 | F (18, 189) = 3.253 | P<0.0001 |
| Time | 749.8 | 9 | 83.31 | F (9, 189) = 26.66 | P<0.0001 |
| Treatment | 254.4 | 2 | 127.2 | F (2, 21) = 4.116 | P=0.0310 |
| Subject | 648.9 | 21 | 30.90 | F (21, 189) = 9.889 | P<0.0001 |
| Residual | 590.5 | 189 | 3.125 |  |  |

| \| Ketamine, Control  ANOVA table \| SS \| DF \| MS \| F (DFn, DFd) \| P value \| \| --- \| --- \| --- \| --- \| --- \| --- \| \| Time x Treatment \| 47.11 \| 9 \| 5.234 \| F (9, 126) = 1.024 \| P=0.4250 \| \| Time \| 124.1 \| 9 \| 13.79 \| F (9, 126) = 2.697 \| P=0.0067 \| \| Treatment \| 18.63 \| 1 \| 18.63 \| F (1, 14) = 0.1441 \| P=0.7100 \| \| Subject \| 1810 \| 14 \| 129.3 \| F (14, 126) = 25.29 \| P<0.0001 \| \| Residual \| 644.3 \| 126 \| 5.114 \|  \|  \| | | | | | | | |  |  |  |  |  |
| --- | --- | --- | --- | --- | --- | --- | --- | --- | --- | --- | --- | --- | --- | --- | --- | --- | --- | --- | --- | --- | --- | --- | --- | --- | --- | --- | --- | --- | --- | --- | --- | --- | --- | --- | --- | --- | --- | --- | --- | --- | --- | --- | --- | --- | --- | --- | --- | --- |
| NLX-101, Control | | | | | | | |  |  |  |  |  |
| \| \| ANOVA table \| SS \| DF \| MS \| F (DFn, DFd) \| P value \| \| --- \| --- \| --- \| --- \| --- \| --- \| \| Time x Treatment \| 91.96 \| 18 \| 5.109 \| F (18, 189) = 0.8595 \| P=0.6280 \| \| Time \| 83.99 \| 9 \| 9.332 \| F (9, 189) = 1.570 \| P=0.1268 \| \| Treatment \| 57.61 \| 2 \| 28.81 \| F (2, 21) = 0.2203 \| P=0.8041 \| \| Subject \| 2746 \| 21 \| 130.8 \| F (21, 189) = 22.00 \| P<0.0001 \| \| Residual \| 1123 \| 189 \| 5.944 \|  \|  \| \| \| --- \| --- \| --- \| --- \| --- \| --- \| --- \| --- \| --- \| --- \| --- \| --- \| --- \| --- \| --- \| --- \| --- \| --- \| --- \| --- \| --- \| --- \| --- \| --- \| --- \| --- \| --- \| --- \| --- \| --- \| --- \| --- \| --- \| --- \| --- \| --- \| --- \| \| NLX-204, Control \| | | | | | | | |  |  |  |  |  |
| ANOVA table | SS | DF | MS | F (DFn, DFd) | P value |  |  |  |  |  |  |  |
| Time x Treatment | 46.61 | 18 | 2.589 | F (18, 189) = 0.4343 | P=0.9789 |  |  |  |  |  |  |  |
| Time | 60.35 | 9 | 6.706 | F (9, 189) = 1.125 | P=0.3470 |  |  |  |  |  |  |  |
| Treatment | 129.4 | 2 | 64.70 | F (2, 21) = 0.4029 | P=0.6734 |  |  |  |  |  |  |  |
| Subject | 3372 | 21 | 160.6 | F (21, 189) = 26.94 | P<0.0001 |  |  |  |  |  |  |  |
| Residual | 1127 | 189 | 5.962 |  |  |  |  |  |  |  |  |  |

**Two Way Analysis of Variance**

**Data source:** NOR index Wistar Day 3

Dependent Variable: Ketamine

**Source of Variation DF SS MS F P**

Stress 1 0.371 0.371 9.245 0.005

Treatment 1 0.547 0.547 13.615 <0.001

Stress x Treatment 1 0.587 0.587 14.612 <0.001

Residual 28 1.125 0.0402

Total 31 2.631 0.0849

Dependent Variable: NLX-101

**Source of Variation DF SS MS F P**

Stress 1 0.304 0.304 7.267 0.010

Treatment 2 0.420 0.210 5.011 0.011

Stress x Treatment 2 0.670 0.335 8.001 0.001

Residual 42 1.760 0.0419

Total 47 3.155 0.0671

Dependent Variable: NLX-204

**Source of Variation DF SS MS F P**

Stress 1 0.527 0.527 14.681 <0.001

Treatment 2 0.300 0.150 4.171 0.022

Stress x Treatment 2 0.505 0.252 7.025 0.002

Residual 42 1.509 0.0359

Total 47 2.841 0.0604

**Data source:** NOR index Wistar Day 17

Dependent Variable: Ketamine

**Source of Variation DF SS MS F P**

Stress 1 0.117 0.117 2.472 0.127

Treatment 1 0.294 0.294 6.202 0.019

Stress x Treatment 1 0.485 0.485 10.241 0.003

Residual 28 1.326 0.0474

Total 31 2.222 0.0717

Dependent Variable: NLX-101

**Source of Variation DF SS MS F P**

Stress 1 0.0966 0.0966 3.914 0.054

Treatment 2 0.510 0.255 10.331 <0.001

Stress x Treatment 2 0.468 0.234 9.486 <0.001

Residual 42 1.036 0.0247

Total 47 2.111 0.0449

Dependent Variable: NLX-204

**Source of Variation DF SS MS F P**

Stress 1 0.172 0.172 5.682 0.022

Treatment 2 0.291 0.145 4.792 0.013

Stress x Treatment 2 0.367 0.184 6.057 0.005

Residual 42 1.274 0.0303

Total 47 2.104 0.0448

| Two-way ANOVA |  |  |  |  |  |
| --- | --- | --- | --- | --- | --- |
| Loco NOR Wistar Day 3 |  |  |  |  |  |
| Ketamine |  |  |  |  |  |
|  |  |  |  |  |  |
| ANOVA table | SS | DF | MS | F (DFn, DFd) | P value |
| Interaction | 214.2 | 1 | 214.2 | F (1, 28) = 0.2021 | P=0.6565 |
| Stress | 273.3 | 1 | 273.3 | F (1, 28) = 0.2578 | P=0.6156 |
| Treatment | 588.2 | 1 | 588.2 | F (1, 28) = 0.5549 | P=0.4625 |
| Residual | 29683 | 28 | 1060 |  |  |

NLX-101

| ANOVA table | SS | DF | MS | F (DFn, DFd) | P value |
| --- | --- | --- | --- | --- | --- |
| Interaction | 1644 | 2 | 822.1 | F (2, 42) = 1.238 | P=0.3002 |
| Stress | 0.3468 | 1 | 0.3468 | F (1, 42) = 0.0005224 | P=0.9819 |
| Treatment | 389.5 | 2 | 194.7 | F (2, 42) = 0.2933 | P=0.7473 |
| Residual | 27885 | 42 | 663.9 |  |  |

NLX-204

| ANOVA table | SS | DF | MS | F (DFn, DFd) | P value |
| --- | --- | --- | --- | --- | --- |
| Interaction | 2197 | 2 | 1098 | F (2, 42) = 1.944 | P=0.1558 |
| Stress | 53.59 | 1 | 53.59 | F (1, 42) = 0.09485 | P=0.7596 |
| Treatment | 254.1 | 2 | 127.1 | F (2, 42) = 0.2249 | P=0.7996 |
| Residual | 23731 | 42 | 565.0 |  |  |

| Loco NOR Wistar Day 17 |  |  |  |  |  |
| --- | --- | --- | --- | --- | --- |
| Ketamine |  |  |  |  |  |
| ANOVA table | SS | DF | MS | F (DFn, DFd) | P value |
| Interaction | 158.4 | 1 | 158.4 | F (1, 28) = 0.1146 | P=0.7375 |
| Stress | 89.78 | 1 | 89.78 | F (1, 28) = 0.06494 | P=0.8007 |
| Rétament | 1362 | 1 | 1362 | F (1, 28) = 0.9855 | P=0.3294 |
| Residual | 38711 | 28 | 1383 |  |  |

| NLX-101 |  |  |  |  |  |
| --- | --- | --- | --- | --- | --- |
| ANOVA table | SS | DF | MS | F (DFn, DFd) | P value |
| Interaction | 2777 | 2 | 1389 | F (2, 42) = 1.869 | P=0.1669 |
| Stress | 67.21 | 1 | 67.21 | F (1, 42) = 0.09046 | P=0.7651 |
| Treatment | 3658 | 2 | 1829 | F (2, 42) = 2.462 | P=0.0975 |
| Residual | 31207 | 42 | 743.0 |  |  |

| NLX-204 |  |  |  |  |  |
| --- | --- | --- | --- | --- | --- |
| ANOVA table | SS | DF | MS | F (DFn, DFd) | P value |
| Interaction | 1904 | 2 | 952.1 | F (2, 42) = 1.172 | P=0.3197 |
| Stress | 0.01333 | 1 | 0.01333 | F (1, 42) = 1.641e-005 | P=0.9968 |
| Treatment | 4381 | 2 | 2190 | F (2, 42) = 2.696 | P=0.0791 |
| Residual | 34125 | 42 | 812.5 |  |  |

**Two Way Analysis of Variance**

**Data source:** EPM Wistar Day 2

Dependent Variable: Ketamine

**Source of Variation DF SS MS F P**

Stress 1 2491.003 2491.003 8.848 0.006

Treatment 1 185.281 185.281 0.658 0.424

Stress x Treatment 1 1046.531 1046.531 3.717 0.064

Residual 28 7882.903 281.532

Total 31 11605.719 374.378

Dependent Variable: NLX-101

**Source of Variation DF SS MS F P**

Stress 1 2811.120 2811.120 13.013 <0.001

Treatment 2 226.500 113.250 0.524 0.596

Stress x Treatment 2 1153.185 576.593 2.669 0.081

Residual 42 9072.889 216.021

Total 47 13263.694 282.206

Dependent Variable: NLX-204

**Source of Variation DF SS MS F P**

Stress 1 1463.021 1463.021 6.245 0.016

Treatment 2 920.727 460.363 1.965 0.153

Stress x Treatment 2 2178.792 1089.396 4.650 0.015

Residual 42 9839.847 234.282

Total 47 14402.387 306.434

**Data source:** EPM Wistar Day 16

Dependent Variable: Ketamine

**Source of Variation DF SS MS F P**

Stress 1 3240.125 3240.125 20.092 <0.001

Treatment 1 455.014 455.014 2.822 0.104

Stress x Treatment 1 1662.722 1662.722 10.311 0.003

Residual 28 4515.417 161.265

Total 31 9873.278 318.493

Dependent Variable: NLX-101

**Source of Variation DF SS MS F P**

Stress 1 6525.558 6525.558 30.850 <0.001

Treatment 2 519.616 259.808 1.228 0.303

Stress x Treatment 2 762.977 381.488 1.803 0.177

Residual 42 8884.181 211.528

Total 47 16692.331 355.156

Dependent Variable: NLX-204

**Source of Variation DF SS MS F P**

Stress 1 1610.083 1610.083 8.871 0.005

Treatment 2 1136.949 568.475 3.132 0.054

Stress x Treatment 2 3561.542 1780.771 9.811 <0.001

Residual 42 7623.194 181.505

Total 47 13931.769 296.421

**Two Way Analysis of Variance**

**Data source:** Body weight Wistar Day 15

Dependent Variable: Ketamine

**Source of Variation DF SS MS F P**

Stress 1 2628.125 2628.125 3.896 0.058

Treatment 1 1653.125 1653.125 2.451 0.129

Stress x Treatment 1 703.125 703.125 1.042 0.316

Residual 28 18887.500 674.554

Total 31 23871.875 770.060

Dependent Variable: NLX-101

**Source of Variation DF SS MS F P**

Stress 1 8.333 8.333 0.0134 0.908

Treatment 2 1029.167 514.583 0.827 0.444

Stress x Treatment 2 379.167 189.583 0.305 0.739

Residual 42 26125.000 622.024

Total 47 27541.667 585.993

Dependent Variable: NLX-204

**Source of Variation DF SS MS F P**

Stress 1 2775.521 2775.521 4.576 0.038

Treatment 2 451.042 225.521 0.372 0.692

Stress x Treatment 2 1001.042 500.521 0.825 0.445

Residual 42 25471.875 606.473

Total 47 29699.479 631.904

**Data source:** Body weight Wistar Week 4

Dependent Variable: Ketamine

**Source of Variation DF SS MS F P**

Stress 1 1725.781 1725.781 1.689 0.204

Treatment 1 2719.531 2719.531 2.662 0.114

Stress x Treatment 1 1313.281 1313.281 1.286 0.266

Residual 28 28603.125 1021.540

Total 31 34361.719 1108.443

Dependent Variable: NLX-101

**Source of Variation DF SS MS F P**

Stress 1 208.333 208.333 0.265 0.609

Treatment 2 1884.375 942.188 1.200 0.311

Stress x Treatment 2 219.792 109.896 0.140 0.870

Residual 42 32968.750 784.970

Total 47 35281.250 750.665

Dependent Variable: NLX-204

**Source of Variation DF SS MS F P**

Stress 1 1200.000 1200.000 1.423 0.240

Treatment 2 2126.042 1063.021 1.261 0.294

Stress x Treatment 2 1053.125 526.563 0.624 0.540

Residual 42 35418.750 843.304

Total 47 39797.917 846.764

**Wistar-Kyoto rats**

**Three Way Analysis of Variance, sucrose intake**

**Data source:** Ketamine

**Source of Variation DF SS MS F P**

Stress 1 983.924 983.924 104.800 <0.001

Treatment 1 173.873 173.873 18.520 <0.001

Time 9 344.948 38.328 4.082 <0.001

Stress x Treatment 1 130.280 130.280 13.876 <0.001

Stress x Time 9 142.211 15.801 1.683 0.093

Treatment x Time 9 141.328 15.703 1.673 0.095

Stress x Treatment x Time 9 84.735 9.415 1.003 0.438

Residual 280 2628.804 9.389

Total 319 4630.102 14.514

**Data source:** NLX-101

**Source of Variation DF SS MS F P**

Stress 1 1539.042 1539.042 150.653 <0.001

Treatment 2 179.859 89.929 8.803 <0.001

Time 9 356.762 39.640 3.880 <0.001

Stress x Treatment 2 114.939 57.469 5.626 0.004

Stress x Time 9 402.179 44.687 4.374 <0.001

Treatment x Time 18 110.525 6.140 0.601 0.899

Stress x Treatment x Time 18 135.537 7.530 0.737 0.773

Residual 420 4290.632 10.216

Total 479 7129.474 14.884

**Data source:** NLX 204

**Source of Variation DF SS MS F P**

Stress 1 1850.795 1850.795 196.115 <0.001

Treatment 2 269.484 134.742 14.278 <0.001

Time 9 276.376 30.708 3.254 <0.001

Stress x Treatment 2 44.748 22.374 2.371 0.095

Stress x Time 9 296.894 32.988 3.496 <0.001

Treatment x Time 18 213.227 11.846 1.255 0.214

Stress x Treatment x Time 18 78.075 4.337 0.460 0.973

Residual 420 3963.672 9.437

Total 479 6993.271 14.600

| Two-way RM ANOVA,  **sucrose intake** |  |
| --- | --- |
|  |  |
| Ketamine, CMS |  |

| ANOVA table | SS | DF | MS | F (DFn, DFd) | P value |
| --- | --- | --- | --- | --- | --- |
| Time x Treatment | 194.0 | 9 | 21.56 | F (9, 126) = 5.344 | P<0.0001 |
| Time | 404.7 | 9 | 44.96 | F (9, 126) = 11.15 | P<0.0001 |
| Treatment | 305.0 | 1 | 305.0 | F (1, 14) = 12.13 | P=0.0037 |
| Subject | 351.9 | 14 | 25.14 | F (14, 126) = 6.232 | P<0.0001 |
| Residual | 508.2 | 126 | 4.034 |  |  |

| NLX-101, CMS |  |  |  |  |  |
| --- | --- | --- | --- | --- | --- |
| ANOVA table | SS | DF | MS | F (DFn, DFd) | P value |
| Time x Treatment | 183.9 | 18 | 10.21 | F (18, 189) = 3.184 | P<0.0001 |
| Time | 657.3 | 9 | 73.03 | F (9, 189) = 22.76 | P<0.0001 |
| Treatment | 284.3 | 2 | 142.2 | F (2, 21) = 5.681 | P=0.0107 |
| Subject | 525.5 | 21 | 25.03 | F (21, 189) = 7.800 | P<0.0001 |
| Residual | 606.4 | 189 | 3.208 |  |  |

| NLX-204, CMS |  |  |  |  |  |
| --- | --- | --- | --- | --- | --- |
| ANOVA table | SS | DF | MS | F (DFn, DFd) | P value |
| Time x Treatment | 169.1 | 18 | 9.395 | F (18, 189) = 2.299 | P=0.0029 |
| Time | 526.3 | 9 | 58.48 | F (9, 189) = 14.31 | P<0.0001 |
| Treatment | 255.4 | 2 | 127.7 | F (2, 21) = 5.890 | P=0.0093 |
| Subject | 455.4 | 21 | 21.68 | F (21, 189) = 5.305 | P<0.0001 |
| Residual | 772.5 | 189 | 4.087 |  |  |

| Ketamine, Control | | | | |  |  |  |  |  | |
| --- | --- | --- | --- | --- | --- | --- | --- | --- | --- | --- |
| ANOVA table | SS | DF | MS | F (DFn, DFd) | P value | | | | |  |
| Time x Treatment | 32.29 | 9 | 3.588 | F (9, 126) = 0.5888 | P=0.8043 | | | | |  |
| Time | 84.22 | 9 | 9.358 | F (9, 126) = 1.536 | P=0.1424 | | | | |  |
| Treatment | 1.640 | 1 | 1.640 | F (1, 14) = 0.02298 | P=0.8817 | | | | |  |
| Subject | 999.1 | 14 | 71.36 | F (14, 126) = 11.71 | P<0.0001 | | | | |  |
| Residual | 767.8 | 126 | 6.094 |  |  | | | | |  |

| NLX-101, Control | |  | |  | |  | |  | |  | |
| --- | --- | --- | --- | --- | --- | --- | --- | --- | --- | --- | --- |
| ANOVA table | | SS | | DF | | MS | | F (DFn, DFd) | | P value | |
| Time x Treatment | | 62.31 | | 18 | | 3.462 | | F (18, 189) = 0.5659 | | P=0.9204 | |
| Time | | 102.6 | | 9 | | 11.40 | | F (9, 189) = 1.863 | | P=0.0598 | |
| Treatment | | 11.33 | | 2 | | 5.663 | | F (2, 21) = 0.05946 | | P=0.9424 | |
| Subject | | 2000 | | 21 | | 95.24 | | F (21, 189) = 15.57 | | P<0.0001 | |
| Residual | | 1156 | | 189 | | 6.117 | |  | |  | |
| NLX-204, Control | |  | |  | |  | |  | |  | |

| ANOVA table | SS | DF | MS | F (DFn, DFd) | P value |
| --- | --- | --- | --- | --- | --- |
| Time x treatment | 123.3 | 18 | 6.851 | F (18, 189) = 0.9724 | P=0.4933 |
| Time | 46.89 | 9 | 5.210 | F (9, 189) = 0.7395 | P=0.6724 |
| treatment | 60.03 | 2 | 30.01 | F (2, 21) = 0.4501 | P=0.6436 |
| Subject | 1400 | 21 | 66.68 | F (21, 189) = 9.464 | P<0.0001 |
| Residual | 1332 | 189 | 7.045 |  |  |

**Two Way Analysis of Variance**

**Data source:** Nor index W-K Day 3

Dependent Variable: Ketamine

**Source of Variation DF SS MS F P**

Stress 1 0.126 0.126 1.924 0.176

Treatment 1 0.410 0.410 6.257 0.018

Stress x Treatment 1 0.525 0.525 8.012 0.009

Residual 28 1.836 0.0656

Total 31 2.898 0.0935

Dependent Variable: NLX-101

**Source of Variation DF SS MS F P**

Stress 1 0.273 0.273 3.040 0.089

Treatment 2 0.545 0.273 3.034 0.059

Stress x Treatment 2 0.432 0.216 2.405 0.103

Residual 42 3.773 0.0898

Total 47 5.023 0.107

Dependent Variable: NLX-204

**Source of Variation DF SS MS F P**

Stress 1 0.141 0.141 1.903 0.175

Treatment 2 0.600 0.300 4.044 0.025

Stress x Treatment 2 0.487 0.244 3.286 0.047

Residual 42 3.114 0.0741

Total 47 4.342 0.0924

**Data source:** NOR index W-K Day 17

Dependent Variable: Ketamine

**Source of Variation DF SS MS F P**

Stress 1 0.221 0.221 2.561 0.121

Treatment 1 0.223 0.223 2.588 0.119

Stress x Treatment 1 0.175 0.175 2.035 0.165

Residual 28 2.412 0.0862

Total 31 3.031 0.0978

Dependent Variable: NLX-101

**Source of Variation DF SS MS F P**

Stress 1 0.0110 0.0110 0.178 0.675

Treatment 2 0.630 0.315 5.110 0.010

Stress x Treatment 2 0.494 0.247 4.007 0.026

Residual 42 2.589 0.0616

Total 47 3.723 0.0792

Dependent Variable: NLX-204

**Source of Variation DF SS MS F P**

Stress 1 0.0399 0.0399 0.374 0.544

Treatment 2 0.437 0.218 2.049 0.142

Stress x Treatment 2 0.398 0.199 1.868 0.167

Residual 42 4.477 0.107

Total 47 5.352 0.114

| Loco NOR W-K Day 3  Ketamine  ANOVA table | SS | DF | MS | F (DFn, DFd) | P value |
| --- | --- | --- | --- | --- | --- |
| Interaction | 13.52 | 1 | 13.52 | F (1, 28) = 0.03806 | P=0.8467 |
| Stress | 1040 | 1 | 1040 | F (1, 28) = 2.927 | P=0.0982 |
| Treatment | 6.480 | 1 | 6.480 | F (1, 28) = 0.01824 | P=0.8935 |
| Residual | 9947 | 28 | 355.2 |  |  |

| NLX-101  ANOVA table | SS | DF | MS | F (DFn, DFd) | P value |
| --- | --- | --- | --- | --- | --- |
| Interaction | 187.8 | 2 | 93.88 | F (2, 42) = 0.2780 | P=0.7587 |
| Stress | 657.1 | 1 | 657.1 | F (1, 42) = 1.946 | P=0.1704 |
| treatment | 17.36 | 2 | 8.680 | F (2, 42) = 0.02570 | P=0.9746 |
| Residual | 14183 | 42 | 337.7 |  |  |

NLX-204

| ANOVA table | SS | DF | MS | F (DFn, DFd) | P value |
| --- | --- | --- | --- | --- | --- |
| Interaction | 99.23 | 2 | 49.61 | F (2, 42) = 0.1307 | P=0.8778 |
| Stress | 894.4 | 1 | 894.4 | F (1, 42) = 2.357 | P=0.1322 |
| Treatment | 1411 | 2 | 705.7 | F (2, 42) = 1.860 | P=0.1683 |
| Residual | 15940 | 42 | 379.5 |  |  |

Loco NOR W-K Day 17

Ketamine

| ANOVA table | SS | DF | MS | F (DFn, DFd) | P value |
| --- | --- | --- | --- | --- | --- |
| Interaction | 10.58 | 1 | 10.58 | F (1, 28) = 0.03620 | P=0.8505 |
| Stress | 2353 | 1 | 2353 | F (1, 28) = 8.052 | P=0.0084 |
| Treatment | 1405 | 1 | 1405 | F (1, 28) = 4.806 | P=0.0368 |
| Residual | 8183 | 28 | 292.2 |  |  |

NLX-101

| ANOVA table | SS | DF | MS | F (DFn, DFd) | P value |
| --- | --- | --- | --- | --- | --- |
| Interaction | 656.6 | 2 | 328.3 | F (2, 42) = 0.7545 | P=0.4765 |
| Stress | 491.5 | 1 | 491.5 | F (1, 42) = 1.130 | P=0.2939 |
| Treatment | 1373 | 2 | 686.5 | F (2, 42) = 1.578 | P=0.2185 |
| Residual | 18276 | 42 | 435.1 |  |  |

NLX-204

| ANOVA table | SS | DF | MS | F (DFn, DFd) | P value |
| --- | --- | --- | --- | --- | --- |
| Interaction | 256.5 | 2 | 128.3 | F (2, 42) = 0.5952 | P=0.5560 |
| Stress | 3241 | 1 | 3241 | F (1, 42) = 15.04 | P=0.0004 |
| Treatment | 3994 | 2 | 1997 | F (2, 42) = 9.269 | P=0.0005 |
| Residual | 9050 | 42 | 215.5 |  |  |

**Two Way Analysis of Variance**

**Data source:** EPM W-K Day 2

Dependent Variable: Ketamine

**Source of Variation DF SS MS F P**

Stress 1 750.781 750.781 12.069 0.002

Treatment 1 129.337 129.337 2.079 0.160

Stress x Treatment 1 255.003 255.003 4.099 0.053

Residual 28 1741.792 62.207

Total 31 2876.913 92.804

Dependent Variable: NLX-101

**Source of Variation DF SS MS F P**

Stress 1 1213.370 1213.370 5.740 0.021

Treatment 2 1474.796 737.398 3.489 0.040

Stress x Treatment 2 175.130 87.565 0.414 0.663

Residual 42 8877.583 211.371

Total 47 11740.880 249.806

Dependent Variable: NLX-204

**Source of Variation DF SS MS F P**

Stress 1 460.454 460.454 2.038 0.161

Treatment 2 1843.352 921.676 4.080 0.024

Stress x Treatment 2 793.130 396.565 1.755 0.185

Residual 42 9488.250 225.911

Total 47 12585.185 267.770

**Data source:** EPM W-K Day 16

Dependent Variable: Ketamine

**Source of Variation DF SS MS F P**

Stress 1 31.337 31.337 0.321 0.576

Treatment 1 19.531 19.531 0.200 0.658

Stress x Treatment 1 432.670 432.670 4.427 0.044

Residual 28 2736.264 97.724

Total 31 3219.802 103.865

Dependent Variable: NLX-101

**Source of Variation DF SS MS F P**

Stress 1 6.750 6.750 0.0518 0.821

Treatment 2 152.019 76.009 0.583 0.563

Stress x Treatment 2 742.167 371.083 2.847 0.069

Residual 42 5474.361 130.342

Total 47 6375.296 135.645

Dependent Variable: NLX-204

**Source of Variation DF SS MS F P**

Stress 1 132.225 132.225 0.395 0.533

Treatment 2 877.588 438.794 1.312 0.280

Stress x Treatment 2 976.144 488.072 1.459 0.244

Residual 42 14046.708 334.445

Total 47 16032.664 341.121

**Two Way Analysis of Variance**

**Data source:** Body weight W-K Day 15

Dependent Variable: Ketamine

**Source of Variation DF SS MS F P**

Stress 1 2112.500 2112.500 5.471 0.027

Treatment 1 1800.000 1800.000 4.661 0.040

Stress x Treatment 1 12.500 12.500 0.0324 0.859

Residual 28 10812.500 386.161

Total 31 14737.500 475.403

Dependent Variable: NLX-101

**Source of Variation DF SS MS F P**

Stress 1 1518.750 1518.750 4.593 0.038

Treatment 2 712.500 356.250 1.077 0.350

Stress x Treatment 2 162.500 81.250 0.246 0.783

Residual 42 13887.500 330.655

Total 47 16281.250 346.410

Dependent Variable: NLX-204

**Source of Variation DF SS MS F P**

Stress 1 4800.000 4800.000 14.517 <0.001

Treatment 2 1015.625 507.813 1.536 0.227

Stress x Treatment 2 178.125 89.063 0.269 0.765

Residual 42 13887.500 330.655

Total 47 19881.250 423.005

**Data source:** Body weight W-K Week 4

Dependent Variable: Ketamine

**Source of Variation DF SS MS F P**

Stress 1 2907.031 2907.031 6.985 0.013

Treatment 1 1444.531 1444.531 3.471 0.073

Stress x Treatment 1 38.281 38.281 0.0920 0.764

Residual 28 11653.125 416.183

Total 31 16042.969 517.515

Dependent Variable: NLX-101

**Source of Variation DF SS MS F P**

Stress 1 4125.521 4125.521 13.507 <0.001

Treatment 2 613.542 306.771 1.004 0.375

Stress x Treatment 2 94.792 47.396 0.155 0.857

Residual 42 12828.125 305.432

Total 47 17661.979 375.787

Dependent Variable: NLX-204

**Source of Variation DF SS MS F P**

Stress 1 5742.188 5742.188 14.983 <0.001

Treatment 2 1219.792 609.896 1.591 0.216

Stress x Treatment 2 228.125 114.063 0.298 0.744

Residual 42 16096.875 383.259

Total 47 23286.979 495.468
